# Family Socioeconomic Position and Lung Cancer Risk: A Meta-Analysis and a Mendelian Randomization Study

**DOI:** 10.3389/fpubh.2022.780538

**Published:** 2022-06-06

**Authors:** Xusen Zou, Runchen Wang, Zhao Yang, Qixia Wang, Wenhai Fu, Zhenyu Huo, Fan Ge, Ran Zhong, Yu Jiang, Jiangfu Li, Shan Xiong, Wen Hong, Wenhua Liang

**Affiliations:** ^1^South China University of Technology, School of Public Administration, Guangzhou, China; ^2^Department of Thoracic Oncology and Surgery, China State Key Laboratory of Respiratory Disease and National Clinical Research Center for Respiratory Disease, the First Affiliated Hospital of Guangzhou Medical University, Guangzhou, China; ^3^Nanshan School, Guangzhou Medical University, Guangzhou, China; ^4^Peking University First Hospital, Beijing, China; ^5^First Clinical School, Guangzhou Medical University, Guangzhou, China

**Keywords:** lung cancer risk, socioeconomic position (SEP), meta-analysis, dose response, Mendelian randomization

## Abstract

**Background:**

Family socioeconomic position (SEP) in childhood is an important factor to predict some chronic diseases. However, the association between family SEP in childhood and the risk of lung cancer is not clear.

**Methods:**

A systematic search was performed to explore their relationship. We selected education level, socioeconomic positions of parents and childhood housing conditions to represent an individual family SEP. Hazard ratios (HRs) of lung cancer specific-mortality were synthesized using a random effects model. Two-sample Mendelian randomization (MR) was carried out with summary data from published genome-wide association studies of SEP to assess the possible causal relationship of SEP and risk of lung cancer.

**Results:**

Through meta-analysis of 13 studies, we observed that to compared with the better SEP, the poorer SEP in the childhood was associated with the increased lung cancer risk in the adulthood (HR: 1.25, 95% CI: 1.10 to 1.43). In addition, the dose-response analysis revealed a positive correlation between the poorer SEP and increased lung cancer risk. Same conclusion was reached in MR [(education level) OR 0.50, 95% CI: 0.39 to 0.63; *P* < 0.001].

**Conclusion:**

This study indicates that poor family socioeconomic position in childhood is causally correlated with lung cancer risk in adulthood.

**Systematic Review Registration:**

identifier: 159082.

## Background

The relationship between everyday-life situations in childhood and chronic diseases has received increased attention across a number of disciplines in recent years (1–4). In addition to the genetic effects, the effects of social environment in early life can also impact one's later life (5, 6). Early prediction of high incidence of lung cancer can play an important role in preventing lung cancer-specific death. Early detection and treatment of lung cancer is a promising strategy to reduce lung cancer mortality (7).

SEP is one of the interfering factors of health of family members (8, 9). Family SEP in childhood consists of many conditions, including years of schooling, parents' social status, childhood housing conditions and so on (10, 11). So far, family SEP in childhood is an increasingly important factor to predict chronic diseases. Multiple studies have indicated that there is an association between SEP and the risk of chronic disease (12–15). Some cohort studies have also demonstrated the impact of family SEP in childhood on adult risk of mortality and cancer incidence (16–29). Overall, poor SEP in early years has an impact on adult morbidity and mortality, especially in cardiovascular and gastrointestinal disorders (30, 31). However, there has been little reliable evidence that lung cancer in adulthood may be related to family SEP in childhood (32, 33).

In this study, we sought to investigate the association between SEP in childhood and lung cancer risk in adulthood. A meta-analysis with dose response and a two-sample Mendelian Randomization (MR) were performed using single or multiple single nucleotide polymorphisms (SNPs) as an instrument in instrumental variable analyses by studying known genetic determinants of the exposure variable of interest.

MR uses genetic variants as instrumental variables for assessing causal relationships from observational data (34) and is an established method for probing questions of causality in observational epidemiology (35). MR technique is being extensively applied to estimate the long-term causal effects of various exposures on clinical and epidemiological outcomes using observational data (36). MR is an established approach to evaluate the effect of an exposure on an outcome. Most importantly, unlike main effect MR studies, gene x environment interaction studies are susceptible to confounding factors (37).

## Methods

### Search Strategy and Inclusion Criteria

**Figure 1:**
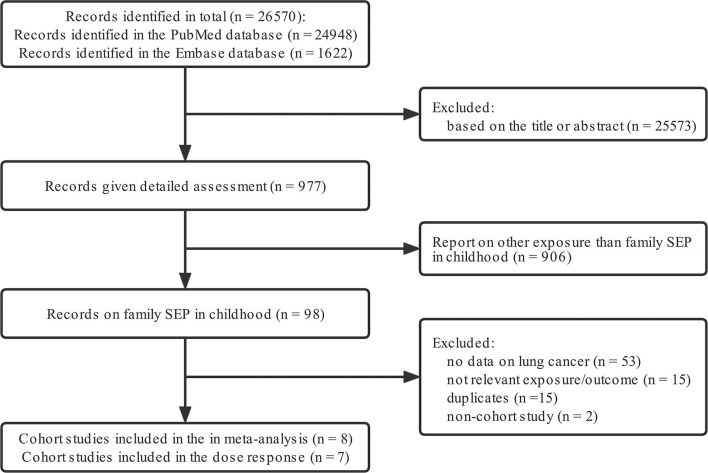
Flow diagram detailing the search strategy and identification of studies used in meta-analysis.

Two investigators (W.R., L.C.) independently searched, PubMed, Web of Science from their inceptions (1966 and 1947, respectively) to June 1, 2020, for cohort studies related to family SEP in childhood and lung cancer in adulthood, without language restrictions. Keywords used were “socioeconomic position (SEP)”, “cohort”, “lung cancer”, “risk”, as well as their Medical Subject Headings (MeSH) terms. Only papers published in English were included. Details of the search terms and inclusion/exclusion criteria are shown in Figure 1.

The inclusion criteria were: (i) cohort study design; (ii) all cases were lung cancer patients, and data were classified according to different SEP, regardless of subtypes of lung cancer; (iii) In addition, the exclusion criteria were: (i) small sample sizes; (ii) non-English publications; (iii) lack of data and necessary information for meta analysis or dose response analysis.

All the included studies were divided into two groups: (I) studies reported adjusted estimates of the hazard ratios (HR) (including relative risk and odds ratios) and 95% confidence interval (95% CI); (II) studies missed data mentioned above. We also included studies that graded data based on parents' financial status and family life in childhood, and overall impact. The quintile or quartile method was incorporated and the first level was used as the reference.

### Data Acquisition Quality Assessment

Data were extracted by two investigators (W.R., H.Z.) independently, and any disagreements came to consensus after discussion. Basic data were recorded from all eligible studies, including the first author's name, publication year, country, study period, period of follow-up, number of lung cancer patients and HR with their 95% Cl. The results were reviewed by two senior investigators (L.W., H.J.).

### Quality Assessment

The quality of the studies was evaluated using a score system that was designed with reference to the Newcastle-Ottawa Scale (NOS) tool (38–40). The system is based on a 0-9 points, with 9 reflecting the highest quality and 0 the lowest. Each point was allocated for the following: (I) representativeness of exposure arm(s), (II) selection of the comparative arm(s), (III) origin of exposure source, (IV) demonstration that outcome of interest was not present at start of study, (V) studies controlling the most important factors, (VI) studies controlling the other main factors, (VII) assessment of outcome with independency, (VIII) adequacy of follow-up length (to assess outcome), (IX) lost to follow-up acceptable (< 10% and reported).

Two researchers (W.R. and G.F.) independently evaluated the methodological quality of each included published cohort study. The results of the quality assessment were used for descriptive purposes, to provide an overall assessment of the quality of the included studies (38, 41–44).

A meta-analysis was performed following the Preferred Reporting Items for Systematic Reviews and Meta-analyses (PRISMA) guidelines and the study protocol of this study is 1,59,082 (45, 46). We collected study-specific HR with 95% Cl for lung cancer to combine the data. Through Cochran's I^2^ statistic, we examined the heterogeneity across studies and statistical heterogeneity was considered if an I^2^ statistic ≥ 50% (47). A random effect model was employed to synthesize hazard ratios (HRs) for lung cancer-specific mortality if high heterogeneity existed (*p* < 0.5, I^2^ > 50%), otherwise a fixed effect model was conducted.

We utilized Funnel plot tests, Begg's test and Egger's test to evaluate the publication bias (48–50). In addition, subgroup analysis was performed according to gender. Sensitivity analysis was conducted by excluding each study in turn to access the stability of results and potential sources of heterogeneity. All statistical manipulation was employed by Stata software (version 12, StataCorp, TX, USA). All *P*-values were 2-tailed; statistical significance was set at *P*-value < 0.05 (51).

The dose-response study was analyzed by Benchmark Dose Software 3.1.2(BMDS 3.1.2) (52). All steps were established under the BMDS guidelines (53–56). Multiple models were selected for analysis, including extra risk assumptions for background and a benchmark response of 5% (39, 40). According to the included studies, the dose response was divided into two categories: family economic status in childhood and childhood housing conditions. At the same time, according to the original data with or without adjustment, it was divided into adjusted and unadjusted group for subgroup analysis and publication bias analysis. In all dose-response models, we used Exponential models and Hill models as the main dose-response observation results, (57) while using other results for verification. Duplicate studies were excluded.

### MR Analysis Using Summary Statistics

The MR method was based on the following three assumptions: (i) the instrumental variables are strongly associated with the family SEP in childhood; (ii) the instrumental variables affect cancer only through their effect on family SEP in childhood and not through any alternative causal pathway; and (iii) the instrumental variables are independent of any confounders (58, 59). To avoid the potential violation of the first assumption, we selected SNPs that meet the threshold of genome-wide significance (*P* < 5 × 10^−8^). Besides, we applied some methods including weighted median and MR–Egger to test the second assumption. Then we evaluated the directional pleiotropy based on the intercept obtained from the MR Egger analysis to satisfy the third assumption (60). We also performed a leave-one-out analysis in which we sequentially omitted one SNP at a time, to evaluate whether the MR estimate was driven or biased by a single SNP.

The analysis was conducted to estimate the effect of family SEP in childhood (*X*) on the risk of lung cancer (*Y*) using genetic variants (*g*), and the causal estimate is equal to *Yg/Xg* (61). For the association between genetic variants and family SEP in childhood (*Xg*), summary data were utilized from published Genome-Wide Association Studies (GWASs), (62–77) including Social Science Genetic Association Consortium (SSGAC) (1,060,068 individuals), MRC Integrative Epidemiology Unit (MRC-IEU) (2,49,790 individuals), UK Biobank (75,244 individuals) and Neale Lab (4,55,571 individuals) (Supplementary Table 1) (78–80). Summary statistics for the association between genetic variants and lung cancer (Yg) are from the International Lung Cancer Consortium (ILCCO) (27,209 individuals) (Supplementary Table 2) (69).

We selected uncorrelated variants to construct the instrumental variables in the two-sample MR analysis. Using both summary statistics for *Yg* and *Xg*, an Inverse Variance-Weighted (IVW) meta-analysis was employed to estimate the effect of genetically determined family SEP in childhood on the risk of lung cancer using the method of Burgess et al. (81):


$${\hat{\beta }}_{IVW}=\frac{\sum {}_{i=1}^{g}X_{g}Y_{g}\sigma_{Yg}{}^{-2}}{\sum {}_{i=1}^{g}X_{g}{}^{2}\sigma_{Yg}{}^{-2}},\;se\left({\hat{\beta }}_{IVW}\right)=\sqrt{\frac{1}{\sum {}_{i=1}^{g}X_{g}{}^{2}\sigma_{Yg}{}^{-2}}}$$

where *Xg* is the beta estimate for the association between the SNP and family SEP in childhood, *Yg* is the beta estimate for the association between the SNP and lung cancer, and σ*Yg* is the standard error for *Yg*. Corresponding HR and 95% CIs were calculated using β^*IVW*^ and se(β^*IVW*^).

In addition, MR-Egger and Weighted Median were also conducted to identify the causality. Ward ratio would only be observed when the first three models cannot be used due to lack of SNPs (82). Leave-one-out analysis was conducted to estimate whether the result was driven by a single SNP. MR-Egger regression was also performed to access the pleiotropy.

## Results

### Family Socioeconomic Position in Childhood and the Risk of Lung Cancer in Adulthood Observational Analyses

A total of eight cohort studies consisting of 2,779,242 cases were included in the final meta-analysis, (16, 17, 20, 23–28) and seven studies were included in the final dose-response analysis (16–22). The age of the included participants ranged from 14 to 74, the majority of the respondents were between the age of 30 to 64 with 2,413 respondents recorded to have died from lung cancer, details about patients characteristics of these studies can be seen in Supplementary Table 3. Supplementary Table 4 presented the result of the quality assessment. Results and study characteristics including name of first author, publication year, country or region, follow-up period, sample size and number of cases or deaths, type of outcome, gender, age range, HRs and 95% CIs were extracted and can be seen in Supplementary Table 5.

The results of quality assessment did not suggest any evidence of publication bias (Supplementary Figures 1, 2) [Unadjusted group: (Begg: 0.673; Egger: 0.182)]; [Adjusted group: (Begg:1; Egger:0.228)]. And sensitivity analysis was performed by sequentially excluding individual studies (Supplementary Figures 3, 4).

**Figure 2:**
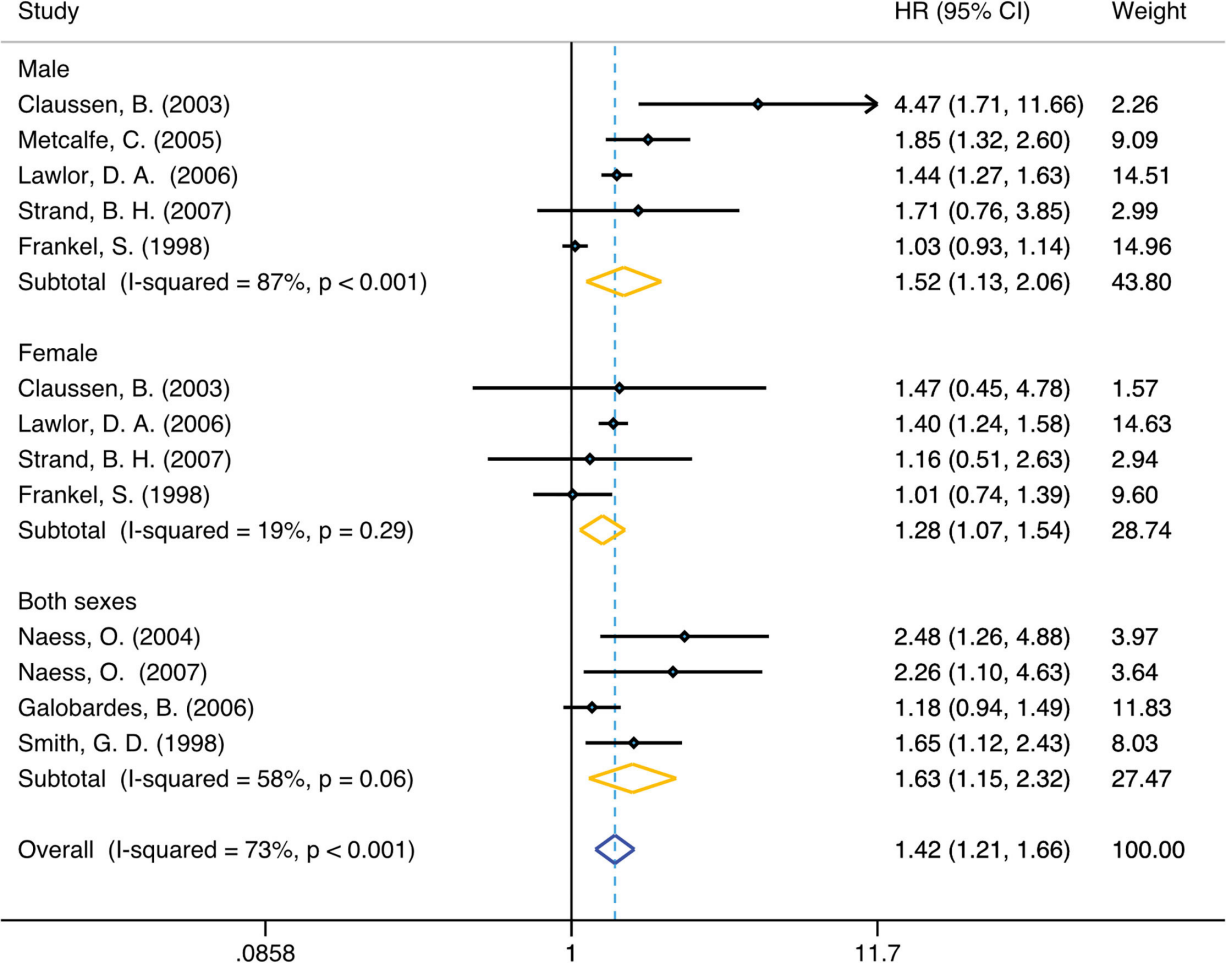
Forest plot of population-based cohort studies of family SEP and lung cancer, stratified by sex (unadjusted group).

**Figure 3:**
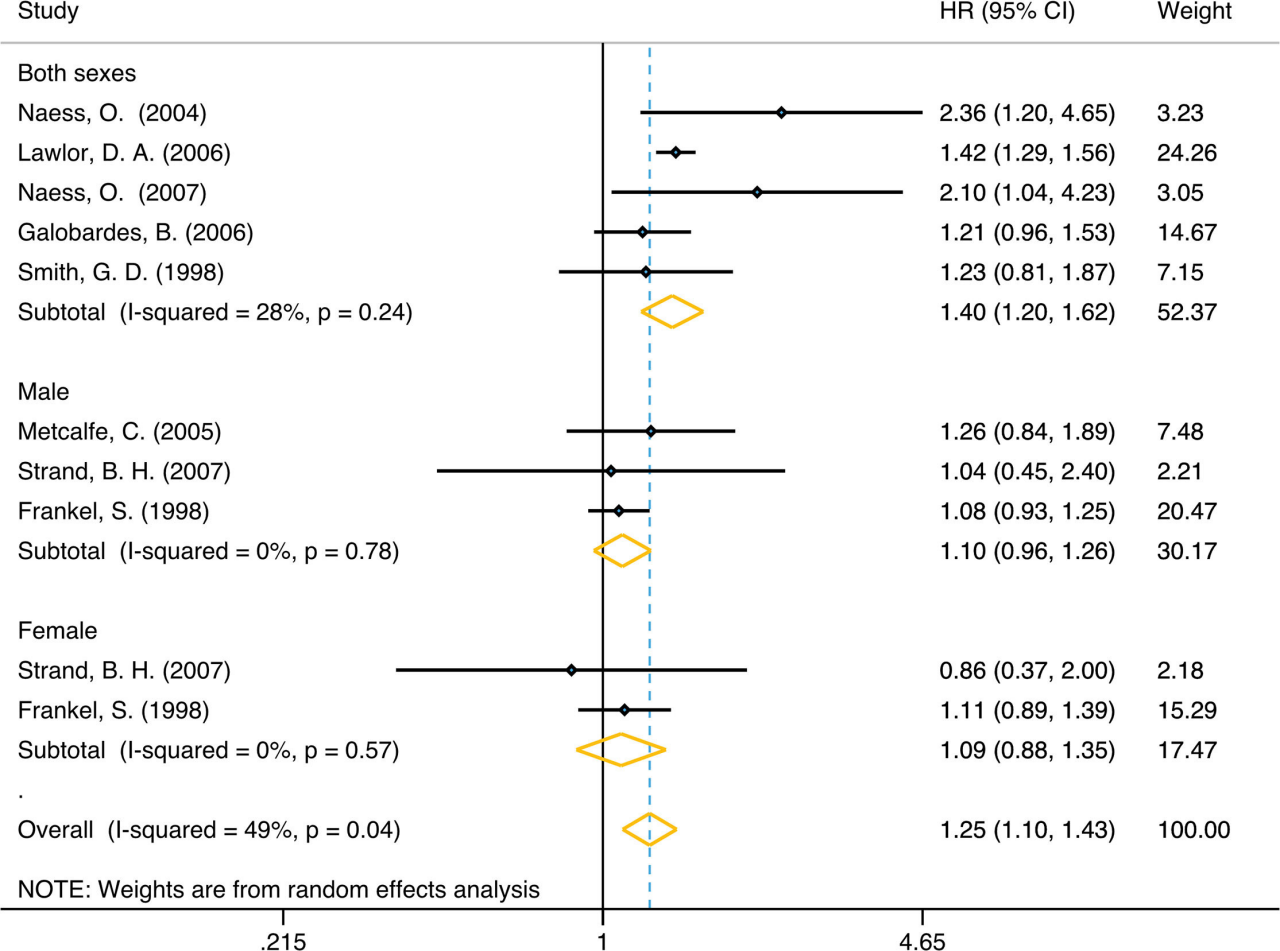
Forest plot of population-based cohort studies of family SEP and lung cancer, stratified by sex (adjusted group).

In unadjusted group, the pooled HR for family economy conditions with best family SEP in childhood group was 1.42 (95% CI, 1.21, 1.66) (I2 = 73.4%, *P* < 0.001) (Table 1, Figure 2). The risk was slightly lower than adjusted group: 1.25 (95% CI, 1.10, 1.42) (I2 = 48.9%, *P* = 0.04) (Table 1, Figure 3). Subgroup analysis based on sex using the provided adjusted original data resulted in a pooled HR of 1.09 (95% CI 0.95, 1.26) (I2 = 0.0%, *p* = 0.78) for male, a pooled HR of 1.10 (95%CI 0.88, 1.35) (I2 = 0.0%, *p* = 0.57) for female, and a pooled HR of 1.39 (95%CI 1.20, 1.62) (I2 = 48.9%, *p* = 0.04) for both sexes. Subgroup analysis based on sex that provided unadjusted original data resulted in a pooled HR of 1.52 (95% CI 1.13, 2.06) (I2 = 87.1%, *p* = 0.006) for male and a pooled HR of 1.28 (95%CI 1.07, 1.54) (I2 = 19.4%, *p* = 0.007) for female and a pooled HR of 1.63 (95%CI 1.15, 2.32) (I2 = 58.7%, *p* = 0.006) for both sexes. The above results was consistent in dose response used the model summary with BMR of 1 Std. Dev. including the family economic conditions of parents and childhood housing conditions (Supplementary Figure 5).

**Table 1:** Pooled estimates from random effects meta-analysis, expressing the HR of risk of lung cancer.

**Outcome**	**Adjusted group**			**Unadjusted group**		
	**HR (95%Cl)**	***P*-value**	**I^**2**^**	**HR (95%Cl)**	***P*-value**	**I^**2**^**
Overall	1.25 (1.10–1.43)	0.04	48.90%	1.42 (1.21–1.66)	<0.001	73.40%
Male	1.10 (0.96–1.26)	0.78	0.00%	1.52 (1.13–2.06)	0.01	87.10%
Female	1.09 (0.88–1.35)	0.57	0.00%	1.28 (1.07–1.54)	0.01	19.40%
Both sexes	1.40 (1.20–1.62)	0.04	48.80%	1.63 (1.15–2.32)	0.01	58.70%

*HR, Hazard Ratio*.

### Genetically Determined Family SEP in Childhood Was Associated With Risk of Lung Cancer in Adulthood

Genetically predicted lower family SEP in childhood was associated with significantly higher odds of lung cancer (Table 2). The causal relationship between length of education and lung cancer was verified first. Using conventional MR analysis, one SD longer education was associated with a 50% lower risk of lung cancer (SSGAC 1: OR 0.50, 95% CI: 0.39, 0.63; *P*: 0.001). Another gene pool with the same exposure factor was also included for verification in our study, showing one SD longer education (genetic predisposition by 4 SNPs) was associated with a 60% lower risk of lung cancer (SSGAC 2: OR 0.40, 95% CI 0.32, 0.50; *p*: 0.001). With a sample size of 27 209, our sample provided sufficient statistical power (> 80%) to detect a causal effect of educational attainment on lung cancer. Power calculations on MR analysis were performed according to Brion et al. (83).

**Table 2:** Mendelian randomization estimates of the associations between family SEP in childhood and risk of lung cancer.

**Outcome**	**Inverse variance weighted**		**Weighted median**		**MR Egger**		**Ward ratio**	
	**OR (95%Cl)**	***P*-value**	**OR (95%Cl)**	***P*-value**	**OR (95%Cl)**	***P*-value**	**OR (95%Cl)**	***P*-value**
The associations between education attainment and risk of lung cancer overall and histological types (Okbay et al.)								
Lung cancer overall	0.49 (0.35–0.69)	<0.01	0.60 (0.39–0.91)	0.02	0.65 (0.11–3.81)	0.63	N/A	N/A
Lung adenocarcinoma	0.64 (0.41–1.02)	0.06	0.66 (0.36–1.20)	0.17	1.35 (0.12–14.80)	0.80	N/A	N/A
Squamous cell lung cancer	0.40 (0.26–0.62)	0.00	0.53 (0.29–0.97)	0.04	0.20 (0.02–1.89)	0.16	N/A	N/A
The associations between education attainment and risk of lung cancer overall and histological types (Lee et al.)								
Lung cancer overall	0.42 (0.28–0.62)	<0.05	0.66 (0.51–0.76)	0.016	0.82 (0.54–1.25)	0.63	N/A	N/A
Lung adenocarcinoma	0.23 (0.08–0.67)	0.06	0.38 (0.19–0.76)	0.17	0.58 (0.20–1.65)	0.80	N/A	N/A
Squamous cell lung cancer	0.40 (0.31–0.52)	<0.01	0.55 (0.46–0.65)	0.04	0.74 (0.57–0.96)	0.16	N/A	N/A
The associations between father's age at death and risk of lung cancer overall and histological types								
Lung cancer overall	N/A	N/A	N/A	N/A	N/A	N/A	2.15E−05 (1.42E−06–3.26E−04)	<0.01
Lung adenocarcinoma	N/A	N/A	N/A	N/A	N/A	N/A	1.77E−05 (3.00E−06–1.04E−04)	<0.01
Squamous cell lung cancer	N/A	N/A	N/A	N/A	N/A	N/A	3.39E−05 (2.33E−06–4.93E−04)	<0.01

*OR, Odds Ratio; N/A, Not available*.

Meanwhile, associations were consistent in sensitivity analyses using weighted median (OR 0.57, 95% CI 0.45, 0.71; *P*: 0.001; Okbay *et al*.) (OR 0.58, 95% CI 0.48, 0.70; *P* < 0.001; Lee *et al*.) and MR-Egger method (OR 0.56, 95% CI 0.17, 1.88; *P*: 0.35; Okbay *et al*.) (OR 0.75, 95% CI 0.60, 0.94; *P*: 0.01; Lee *et al*.), but provided less precise estimates than with conventional MR (IVW method). Nonetheless, their causal estimates were similar in terms of direction and magnitude, and they were unlikely to occur by chance alone. These results were consistent with the hypothesis that genetic pleiotropy does not drive the result.

We also analyzed and observed the relationship between other exposure factors related to family SEP in childhood and lung cancer, and we found that the *P*-values for the intercept were large and the estimates adjusted for pleiotropy suggested null effects in four exposure factors: number of children fathered (Neale Lab): (OR 0.92, 95% CI: 0.29, 2.84; *p*: 0.88); mother's age at death (Neale Lab): (OR 1.00, 95% CI: 0.19, 5.26; *p*: 1.00); and average total household income before tax (MRC-IEU): (OR 0.90, 95% CI: 0.77, 1.04; *p*: 0.156). Nine out of the 15 independent exposure factor gene pools were unable to be analyzed due to the lack of SNPs corresponding to lung cancer.

## Discussion

Based on data from 2,779,242 respondents, we observed that poorer family SEP in childhood means higher lung cancer risk in adulthood compared to controls. The overall dose-response of multiple models also demonstrated this trend. This phenomenon was partially verified by Mendelian randomization of two samples, but the causal relationship between some genes was still unclear. Through meta-analysis, we analyzed both the adjusted (age/adult social class) and unadjusted data, and strengthened the credibility by mutual authentication. The results showed a link between family SEP in childhood and the risk of lung cancer in adulthood in the unadjusted group.

People in poor family economic conditions were more likely to smoke, leading to a higher risk of lung cancer (84). Furthermore, the poor face a variety of problems that may pose other risks including malnutrition, violence, AIDS, and other infectious diseases, which also correlate with lung cancer risk (85, 86). The dose-response curve of family SEP in childhood also contribute to risk. Specifically, the lower the family economic conditions in childhood and the worse the childhood housing conditions, the more likely a person is to develop lung cancer in adulthood. This result has been verified in both the adjusted and unadjusted groups. We selected factors as comprehensive as possible to explore the relationship between family SEP in childhood and the risk of lung cancer in adulthood using two sample MR. Only the data from the official database were used, which made our data more reliable and useful. The results show that there is a clear causal relationship between the length of education and the occurrence of lung cancer. People with low education during childhood were more likely to smoke or be exposed to undesirable living environments, and lack relevant basic medical knowledge. Moreover, Children of parents who smoke perceived casual smoking to be safer and then reported wanting to smoke in response to smoking-related cues more than children of non-smoking parents (87). It should be noted that further research is needed to prove the relationship between father's age of death and lung cancer. On the one hand, although our results showed a strong correlation between SEP and the risk of lung cancer, only one SNP was included. This is too small to calculation of explained variance. This limited the representativeness of the result of father's death of age and lung cancer. On the other hand, early parental death, when a child experiences the loss of a parent before age 18, is one of the most severe stressors and a potential risk factor for adult psychopathology (88). It may cause an increased likelihood of poor habits, for example, smoking, which in turn lead to lung cancer. Worth mentioning, among the results related to education duration, the results of incorporating the genome determined by Okbay et al. were slightly different from the previous results of Zhou et al. (89). This may be explained by these observations: the statistical significance was set as *P*-value < 0.001 instead of 0.01 in the R package used for two-sample MR (34, 90). Because of this, the positive results of our study are more reliable. For the negative result in two-sample Mendelian randomization, we believe that further researcher is required to verify the result. Because the data of the SNPs in the existing studies are still limited, the final results of these studies have not yet been proven.

Three strengths of our study should be highlighted. First, to our knowledge, this is the most comprehensive meta-analysis estimating the relationship between family economic position in childhood and the risk of lung cancer in adulthood. The collection of such large sample data to assess the cancer risk for different family SEP in childhood around the world has not previously been performed. Second, we included adjusted and non-adjusted data for all-factors to mutually verify the dose-response of parents' socioeconomic status and family congestion in childhood. Third, this is the first study to explore the causal relationship between family SEP in childhood and the risk in adulthood by two sample MR. This research offered evidence for researcher focus on epidemiological characters in early detection of lung cancer and clinical staff of early diagnosis of lung cancer.

There are several limitations in our study. First, the studies included for meta-analysis and dose-response studies originated from the UK, Norway, and the Netherlands, there is the risk of introducing potentially heterogeneity. Second, because of the long-term follow-up needs of this study, included studies do not have data from recent years. Third, because the included cohort studies were all-factor epidemiological studies, subgroups of different lung cancer subtypes or specific age could not be established in the meta-analysis. Fourth, in terms of meta-analysis, due to the lack of subgroup data, the results obtained by the adjusted male and female subgroups were not statistically significant. Another potential limitation is that we only included common polymorphisms. Finally, although we have included many different exposure genes to build connections with lung cancer gene pool, some Mendelian randomization results are negative. However, limited SNPs of SEP can lead to false negative result. Further verification is needed to make a precise conclusion for the reason given above.

This research needs to be improved. Cohort results from other countries and expanded observations from lung cancer to all-factor risk are necessary for future studies. Genetic characteristics of continents other than Europe should also be included. Overall, our research confirms our conjecture: the family SEP in childhood is inversely proportional to the risk of lung cancer, which has a positive effect on the early diagnosis and intervention of lung cancer.

## Conclusion

This study indicates that poor family SEP in childhood is a causal risk factor for lung cancer, and thus lung cancer screening should be more heavily considered for these populations. More research is needed to cross-validate these findings.

## Data Availability Statement

The original contributions presented in the study are included in the article/Supplementary Material, further inquiries can be directed to the corresponding authors.

## Author Contributions

XZ and RW: conceptualization and project administration. ZY, XZ, and RW: investigation. ZH, ZY, XZ, YJ, and RW: data curation. ZH: visualization and writing—review and editing. FG, XZ, and RW: formal analysis. FG, ZY, XZ, and RW: writing—original draft. RZ: methodology and software. JL: definition. JL and SX: validation. SX: supervision and funding acquisition. WH: resources. WH and ZH: writing—review and editing. All authors were involved in the conception and design of the study, critically reviewed, and approved the final manuscript.

## Funding

This work was supported by the China National Science Foundation (Grant No. 81871893) and Key Project of Guangzhou Scientific Research Project (Grant No. 201804020030).

## Conflict of Interest

The authors declare that the research was conducted in the absence of any commercial or financial relationships that could be construed as a potential conflict of interest.

## Publisher's Note

All claims expressed in this article are solely those of the authors and do not necessarily represent those of their affiliated organizations, or those of the publisher, the editors and the reviewers. Any product that may be evaluated in this article, or claim that may be made by its manufacturer, is not guaranteed or endorsed by the publisher.

## Supplementary Material

Supplementary Figure 1 Begg funnel plots of population-based cohort studies of family SEP and lung cancer (unadjusted group).

Supplementary Figure 2 Begg funnel plots of population-based cohort studies of family SEP and lung cancer (adjusted group).

Supplementary Figure 3 Sensitivity analysis of population-based cohort studies of family SEP and lung cancer (unadjusted group).

Supplementary Figure 4 Sensitivity analysis of population-based cohort studies of family SEP and lung cancer (adjusted group).

Supplementary Figure 5 Dose response of childhood housing conditions and lung cancer (adjusted group) (top left), family economic conditions and lung cancer (adjusted group) (top right), childhood housing conditions and lung cancer (unadjusted group) (bottom left), family economic conditions and lung cancer (unadjusted group) (bottom right).

Supplementary Table 1 Summary of identified MR gene bank of exposure.

Supplementary Table 2 Summary of identified MR gene bank of outcome.

Supplementary Table 3 Characteristic of the included studies in the meta-analysis.

Supplementary Table 4 The Newcastle-Ottawa quality assessment scale of including studies.

Supplementary Table 5 Data from included cohort studies.

## Abbreviations

CI: Confidence interval
GWASs: Genome-wide association studies
HR: Hazard ratio
ILCCO: International Lung Cancer Consortium
IVW: Inverse Variance-Weighted
MeSH: Medical Subject Headings
MR: Mendelian randomization
OR: Odds ratios
RR: Relative risk
SCLC: Small cell lung cancer
SNP: Single-nucleotide polymorphism
TSMR: Two-sample Mendelian randomization
BMDS: Benchmark Dose Software.
