# Supplementary figures and images for: Family Socioeconomic Position and Lung Cancer Risk: A Meta-Analysis and a Mendelian Randomization Study
Source: Front Public Health. 2022 Jun 6;10:780538. doi: 10.3389/fpubh.2022.780538 (PMC9207765; doi:10.3389/fpubh.2022.780538)

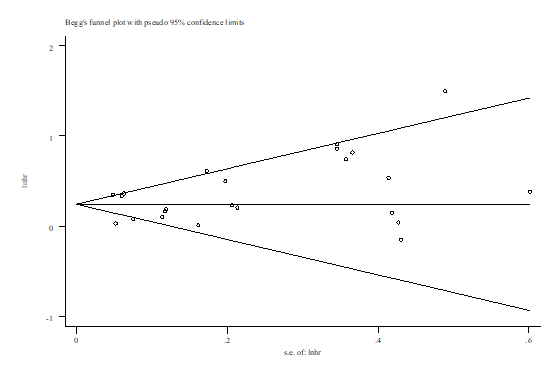

Supplement: Supplementary Figure 1 — Begg funnel plots of population-based cohort studies of family SEP and lung cancer (unadjusted group). [file Image_1.TIF]

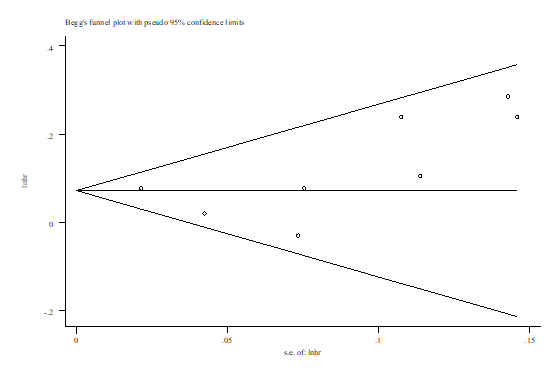

Supplement: Supplementary Figure 2 — Begg funnel plots of population-based cohort studies of family SEP and lung cancer (adjusted group). [file Image_2.TIF]

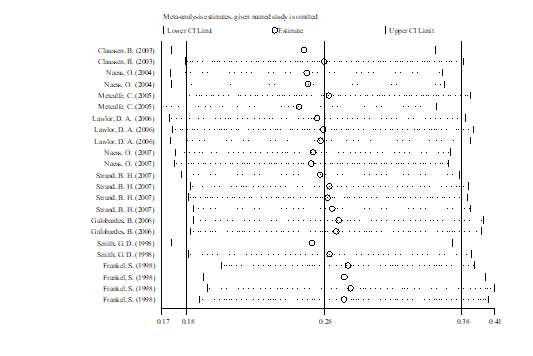

Supplement: Supplementary Figure 3 — Sensitivity analysis of population-based cohort studies of family SEP and lung cancer (unadjusted group). [file Image_3.TIF]

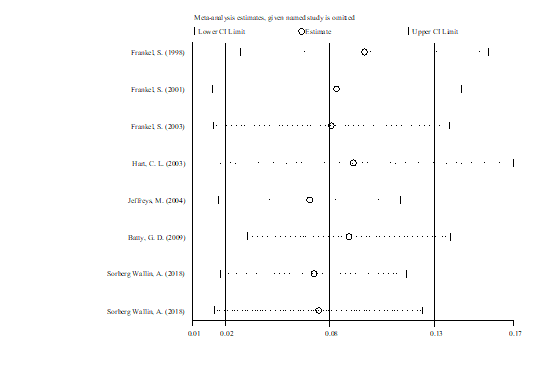

Supplement: Supplementary Figure 4 — Sensitivity analysis of population-based cohort studies of family SEP and lung cancer (adjusted group). [file Image_4.TIF]

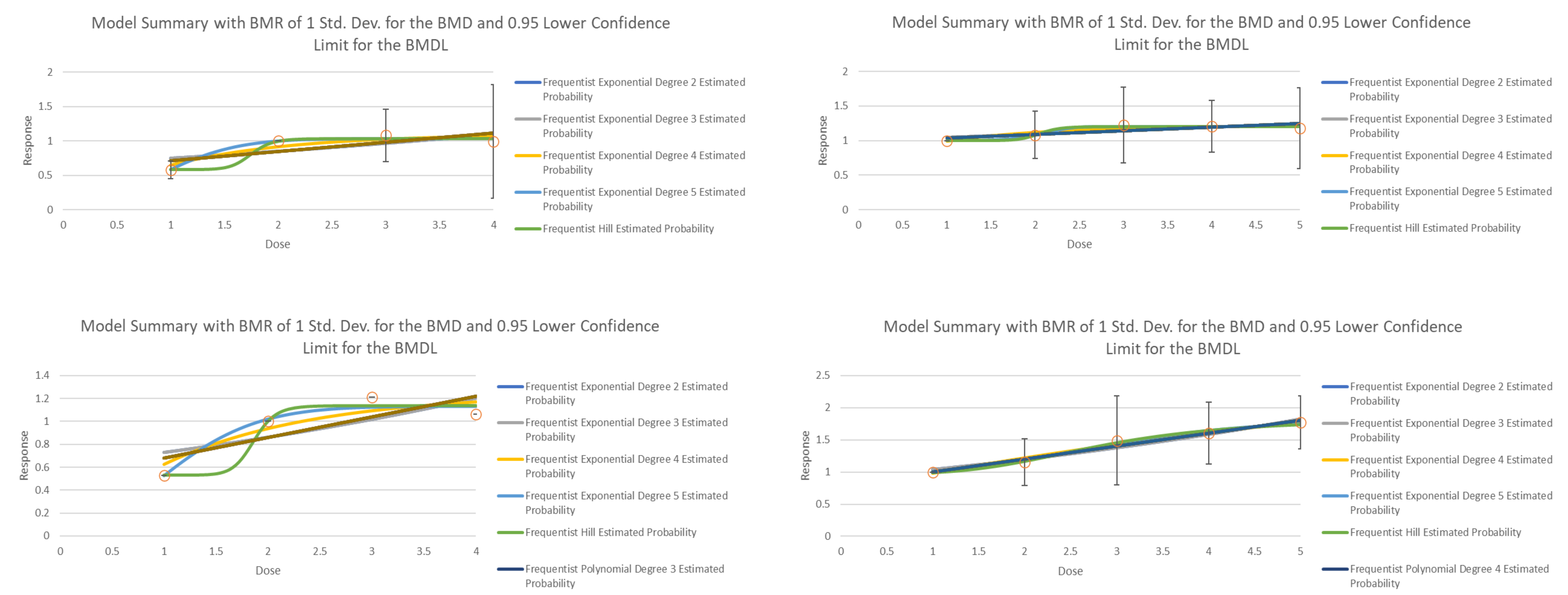

Supplement: Supplementary Figure 5 — Dose response of childhood housing conditions and lung cancer (adjusted group) (top left), family economic conditions and lung cancer (adjusted group) (top right), childhood housing conditions and lung cancer (unadjusted group) (bottom left), family economic conditions and lung cancer (unadjusted group) (bottom right). [file Image_5.TIFF]
