# Supplementary material for: Family Socioeconomic Position and Lung Cancer Risk: A Meta-Analysis and a Mendelian Randomization Study
Source: Front Public Health. 2022 Jun 6;10:780538. doi: 10.3389/fpubh.2022.780538 (PMC9207765; doi:10.3389/fpubh.2022.780538)
Supplement: Supplementary Table 1 — Summary of identified MR gene bank of exposure. [file Data_Sheet_1.DOCX]

**Supplement Table1.** Summary of identified MR gene bank of exposure.

| **ID** |  | **Trait** |  | **Year** |  | **First author** |  | **Consortium** |  | **Sample size** |  | **Number of variants** |  | **population** |  | **Sex** |  | **PMID** |
| --- | --- | --- | --- | --- | --- | --- | --- | --- | --- | --- | --- | --- | --- | --- | --- | --- | --- | --- |
| 1001 |  | Years of schooling |  | 2016 |  | Okbay |  | SSGAC 1 |  | 293723 |  | 8146841 |  | European |  | Males and females |  | 27225129 |
| 1239 |  | Years of schooling |  | 2018 |  | Lee |  | SSGAC 2 |  | 766345 |  | 10101242 |  | European |  | Males and females |  | 30038396 |
| UKB-b:11303 |  | Father's age at death |  | 2018 |  | Ben Elsworth |  | MRC-IEU |  | 341118 |  | 9851867 |  | European |  | Males and females |  | - |
| 1902 |  | Father's age at death |  | 2016 |  | Pilling |  | UK Biobank |  | 75244 |  | 9583643 |  | European |  | Males and females |  | 27015805 |
| UKB-b:12687 |  | Mother's age at death |  | 2018 |  | Ben Elsworth |  | MRC-IEU |  | 273111 |  | 9851867 |  | European |  | Males and females |  | - |
| UKB-a:345 |  | Mother's age at death |  | 2017 |  | Neale |  | Neale Lab |  | 199690 |  | 10894596 |  | European |  | Males and females |  | - |
| 1093 |  | Mother's age at death |  | 2016 |  | Pilling |  | UK Biobank |  | 75244 |  | 9583643 |  | European |  | Males and females |  | 27015805 |
| UKB-b：13183 |  | Adopted as a child |  | 2018 |  | Ben Elsworth |  | MRC-IEU |  | 461805 |  | 9851867 |  | European |  | Males and females |  | - |
| UKB-b:2227 |  | Number of children fathered |  | 2018 |  | Ben Elsworth |  | MRC-IEU |  | 209872 |  | 9851867 |  | European |  | Males and females |  | - |
| UKB-a:304 |  | Number of children fathered |  | 2017 |  | Neale |  | Neale Lab |  | 154888 |  | 10894596 |  | European |  | Males and females |  | - |
| UKB-b:16927 |  | Age of primiparous women at birth of child |  | 2018 |  | Ben Elsworth |  | MRC-IEU |  | 33147 |  | 9851867 |  | European |  | Males and females |  | - |
| UKB-a:33 |  | Breastfed as a baby |  | 2017 |  | Neale |  | Neale Lab |  | 255881 |  | 10894596 |  | European |  | Males and females |  | - |
| UKB-b:13423 |  | Breastfed as a baby |  | 2018 |  | Ben Elsworth |  | MRC-IEU |  | 352094 |  | 9851867 |  | European |  | Males and females |  | - |
| UKB-b:6259 |  | Born in hospital |  | 2018 |  | Ben Elsworth |  | MRC-IEU |  | 463010 |  | 9851867 |  | European |  | Males and females |  | - |
| UKB-b:7408 |  | Average total household income before tax |  | 2018 |  | Ben Elsworth |  | MRC-IEU |  | 397751 |  | 9851867 |  | European |  | Males and females |  | - |
|  |  |  |  |  |  |  |  |  |  |  |  |  |  |  |  |  |  |  |

**Supplement Table2.** Summary of identified MR gene bank of outcome.

| **ID** |  | **Trait** |  | **Year** |  | **First author** |  | **Consortium** |  | **Number of cases** |  | **Number of controls** |  | **Sample size** |  | **Number of variants** |  | **population** |  | **Sex** |  | **PMID** |
| --- | --- | --- | --- | --- | --- | --- | --- | --- | --- | --- | --- | --- | --- | --- | --- | --- | --- | --- | --- | --- | --- | --- |
| 965 |  | Lung adenocarcinoma |  | 2014 |  | Wang Y |  | ILCCO |  | 3442 |  | 14894 |  | 18336 |  | 8881354 |  | European |  | Males and females |  | 24880342 |
| 966 |  | Lung cancer |  | 2014 |  | Wang Y |  | ILCCO |  | 11348 |  | 15861 |  | 27209 |  | 8945893 |  | European |  | Males and females |  | 24880342 |
| 967 |  | Squamous cell lung cancer |  | 2014 |  | Wang Y |  | ILCCO |  | 3275 |  | 15038 |  | 18313 |  | 8893750 |  | European |  | Males and females |  | 24880342 |
|  |  |  |  |  |  |  |  |  |  |  |  |  |  |  |  |  |  |  |  |  |  |  |

**Supplement Table3.** Characteristic of the included studies in the meta-analysis.

| **Author** |  | **Year** |  | **Country** |  | **Sample size** |  | **Number of lung cancer death** |  | **Study period** | | |  | **age range** |
| --- | --- | --- | --- | --- | --- | --- | --- | --- | --- | --- | --- | --- | --- | --- |
|  |  |  |  |  |  |  |  |  |  | **Period 1** |  | **Period 2** |  |  |
| Claussen, B. |  | 2003 |  | Norway |  | 128723 |  | 163 |  | 1960 |  | 1990-1994 |  | 31-55 |
| Naess, O. |  | 2004 |  | Norway |  | 58751 |  | 104 |  | 1960 |  | 1990-1998 |  | 30-54 |
| Metcalfe, C. |  | 2005 |  | British |  | 5577 |  | 255 |  | - |  | 1970-1973 |  | 35-64 |
| Lawlor, D. A. |  | 2006 |  | British |  | 1845716 |  | - |  | 1944-1960 |  | 1980-2001 |  | 20-57 |
| Naess, O. |  | 2007 |  | Norway |  | 55716 |  | 106 |  | 1960 |  | 1990-1998 |  | 30-54 |
| Strand, B. H. |  | 2007 |  | British |  | 613807 |  | 141 |  | 1955-1965 |  | 1990-2001 |  | 25-46 |
| Galobardes, B. |  | 2006 |  | British |  | 11755 |  | 86 |  | 1948-1968 |  | 1998-2005 |  | 30-57 |
| Frankel, S. |  | 1998 |  | British |  | 5645 |  | - |  | 1970-1973 |  | 1980-1998 |  | 35-64 |
| Power, C. |  | 2005 |  | British |  | 11855 |  | 262 |  | 1958 |  | 1991 |  | 14-49 |
| Hart, C. L. |  | 2003 |  | British |  | 5765 |  | 266 |  | - |  | 1970-1973 |  | 35-64 |
| Dedman, D. J. |  | 2001 |  | British |  | 4168 |  | 62 |  | 1937-1939 |  | 1948 |  | - |
| de Kok, I. M. |  | 2008 |  | Netherlands |  | 27020 |  | 189 |  | 1991 |  | 2005 |  | 15-74 |
| Frankel, S. |  | 1998 |  | British |  | 4744 |  | - |  | 1937-1939 |  | 1948 |  | - |
|  |  |  |  |  |  |  |  |  |  |  |  |  |  |  |

**Supplement Table 4.** The Newcastle-Ottawa quality assessment scale of including studies.

| **Studies** | |  | **Selection** | | | | |  | **Comparability** | | | | |  | **Assessment of outcome** | | | | | |  | **Total quality score** |
| --- | --- | --- | --- | --- | --- | --- | --- | --- | --- | --- | --- | --- | --- | --- | --- | --- | --- | --- | --- | --- | --- | --- |
| **Author** | **Year** |  | **Representativeness of exposure arm(s)** |  | **Selection of the comparative arm(s)** |  | **Origin of exposure source** |  | **Demonstration that outcome of interest was not present at start of study** |  | **Studies controlling the most important factors** |  | **Studies controlling the other main factors** |  | **Assessment of outcome with independency** |  | **Adequacy of follow-up length (to assess outcome)** |  | **Lost to follow-up acceptable (less than10% and reported)** |  | |  |
| Claussen, B. | 2003 |  | * |  | * |  | * |  | * |  | * |  | * |  | * |  | * |  |  |  | | 8 |
| Naess, O. | 2004 |  | * |  | * |  | * |  | * |  | * |  | * |  | * |  | * |  |  |  | | 8 |
| Metcalfe, C. | 2005 |  | * |  | * |  | * |  | * |  | * |  | * |  | * |  | * |  |  |  | | 8 |
| Lawlor, D. A. | 2006 |  | * |  | * |  | * |  | * |  | * |  | * |  | * |  | * |  |  |  | | 8 |
| Naess, O. | 2007 |  | * |  | * |  | * |  | * |  | * |  | * |  | * |  | * |  |  |  | | 8 |
| Strand, B. H. | 2007 |  | * |  | * |  | * |  | * |  | * |  | * |  | * |  | * |  |  |  | | 8 |
| Galobardes, B. | 2006 |  | * |  | * |  | * |  | * |  | * |  | * |  | * |  | * |  |  |  | | 8 |
| Frankel, S. | 1998 |  | * |  | * |  | * |  | * |  | * |  | * |  | * |  | - |  |  |  | | 7 |
| Power, C. | 2005 |  | * |  | * |  | * |  | * |  | * |  | * |  | * |  | * |  |  |  | | 8 |
| Hart, C. L. | 2003 |  | * |  | * |  | * |  | * |  | * |  | * |  | * |  | * |  |  |  | | 8 |
| Dedman, D. J. | 2001 |  | * |  | * |  | * |  | * |  | * |  | - |  | * |  | * |  |  |  | | 7 |
| de Kok, I. M. | 2008 |  | * |  | * |  | * |  | * |  | * |  | * |  | * |  | * |  |  |  | | 8 |
| Frankel, S. | 1998 |  | * |  | * |  | * |  | * |  | * |  | - |  | * |  | - |  |  |  | | 6 |
|  |  |  |  |  |  |  |  |  |  |  |  |  |  |  |  |  |  |  |  |  | |  |
|  |  |  |  |  |  |  |  |  |  |  |  |  |  |  |  |  |  |  |  |  | |  |

**Supplement Table 5.** Data from included cohort studies.

| Author |  | Year |  | SEP factors |  | Data classification |  | sex |  | adjusted |  | effect value | | | | | | | | | | | | | | | | | | | | | | | | | | | | | | | | | | | |
| --- | --- | --- | --- | --- | --- | --- | --- | --- | --- | --- | --- | --- | --- | --- | --- | --- | --- | --- | --- | --- | --- | --- | --- | --- | --- | --- | --- | --- | --- | --- | --- | --- | --- | --- | --- | --- | --- | --- | --- | --- | --- | --- | --- | --- | --- | --- | --- |
|  |  |  |  |  |  |  |  |  |  |  |  | Ⅰ |  | 95%Cl | | |  | Ⅱ |  | 95%Cl | | |  | Ⅲ |  | 95%Cl | | |  | Ⅳ |  | 95%Cl | | | |  | Ⅴ |  | 95%Cl | | |  | totall |  | 95%Cl | |  |
| Galobardes, B. |  | 2006 |  | Family economy conditions |  | Y |  | Both sexes |  | n |  | 1 |  |  |  |  |  | 1.0109 |  | 0.606 |  | 4.2 |  | 1.2362 |  | 1.148 |  | 3.147 |  | 1.318 |  | 0.058 |  | 2.578 |  | - |  | - |  | - |  | 1.18 |  | 0.94 |  | 1.49 |  |
|  |  |  |  |  |  |  |  | Both sexes |  | y |  | 1 |  |  |  |  |  | 1.136 |  | 0.467 |  | 2.773 |  | 1.352 |  | 0.357 |  | 5.218 |  | 1.335 |  | 0.349 |  | 5.145 |  | - |  | - |  | - |  | 1.21 |  | 0.96 |  | 1.53 |  |
| Smith, G. D. |  | 1998 |  | Family economy conditions |  | Y |  | Both sexes |  | n |  | 1 |  |  |  |  |  | 1.49 |  | 0.74 |  | 3.02 |  | 2.13 |  | 1.24 |  | 3.68 |  | 1.8 |  | 1.02 |  | 3.17 |  | 1.8 |  | 1.02 |  | 3.17 |  | 1.65 |  | 1.12 |  | 2.43 |  |
|  |  |  |  |  |  |  |  | Both sexes |  | y |  | 1 |  |  |  |  |  | 1.35 |  | 0.67 |  | 2.74 |  | 1.62 |  | 0.92 |  | 2.85 |  | 1.24 |  | 0.68 |  | 2.27 |  | 1.24 |  | 0.68 |  | 2.27 |  | 1.23 |  | 0.81 |  | 1.87 |  |
| Power, C. |  | 2005 |  | Family economy conditions |  | Y |  | Both sexes |  | n |  | 1 |  |  |  |  |  | 1.35 |  | 0.58 |  | 2.06 |  | 1.41 |  | 0.98 |  | 2.05 |  | 1.92 |  | 1.32 |  | 2.81 |  | 1.92 |  | 1.32 |  | 2.81 |  | - |  | - |  | - |  |
|  |  |  |  |  |  |  |  | Both sexes |  | y |  | 1 |  |  |  |  |  | 0.83 |  | 0.44 |  | 1.56 |  | 0.98 |  | 0.67 |  | 1.44 |  | 1.21 |  | 0.81 |  | 1.81 |  | 1.21 |  | 0.81 |  | 1.81 |  | - |  | - |  | - |  |
| de Kok, I. M. |  | 2008 |  | Family economy conditions |  | Y |  | Both sexes |  | N |  | 1 |  |  |  |  |  | 1.02 |  | 0.48 |  | 2.16 |  | 1.19 |  | 0.58 |  | 2.46 |  | 1.37 |  | 0.68 |  | 2.77 |  | 1.59 |  | 0.79 |  | 3.22 |  | - |  | - |  | - |  |
|  |  |  |  |  |  |  |  | Both sexes |  | Y |  | 1 |  |  |  |  |  | 1.02 |  | 0.48 |  | 2.16 |  | 1.19 |  | 0.58 |  | 2.46 |  | 1.37 |  | 0.68 |  | 2.77 |  | 1.59 |  | 0.79 |  | 3.22 |  | - |  | - |  | - |  |
| Hart, C. L. |  | 2003 |  | Family living conditions |  | Y |  | Both sexes |  | y |  | 0.57 |  | 0.3 |  | 1.06 |  | 1 |  |  |  |  |  | 1.11 |  | 0.81 |  | 1.52 |  | 1.06 |  | 0.73 |  | 1.54 |  | 1.65 |  | 1.17 |  | 2.33 |  | - |  | - |  | - |  |
| Dedman, D. J. |  | 2001 |  | Family living conditions |  | Y |  | Both sexes |  | n |  | 0.53 |  | 0.23 |  | 1.21 |  | 1 |  |  |  |  |  | 1.21 |  | 0.69 |  | 2.1 |  | 1.06 |  | 0.55 |  | 2.02 |  | - |  | - |  | - |  | - |  | - |  | - |  |
|  |  |  |  |  |  |  |  | Both sexes |  | y |  | 0.59 |  | 0.25 |  | 1.4 |  | 1 |  |  |  |  |  | 1.05 |  | 0.6 |  | 1.86 |  | 0.93 |  | 0.47 |  | 1.81 |  | - |  | - |  | - |  | - |  | - |  | - |  |
| Claussen, B. |  | 2003 |  | Family economy conditions |  | N |  | male |  | n |  | - |  | - |  | - |  | - |  | - |  | - |  | - |  | - |  | - |  | - |  | - |  | - |  | - |  | - |  | - |  | 4.47 |  | 1.72 |  | 11.7 |  |
|  |  |  |  |  |  |  |  | female |  | n |  | - |  | - |  | - |  | - |  | - |  | - |  | - |  | - |  | - |  | - |  | - |  | - |  | - |  | - |  | - |  | 1.47 |  | 0.45 |  | 4.75 |  |
| Naess, O. |  | 2004 |  | Family economy conditions |  | N |  | Both sexes |  | n |  | - |  | - |  | - |  | - |  | - |  | - |  | - |  | - |  | - |  | - |  | - |  | - |  | - |  | - |  | - |  | 2.48 |  | 1.26 |  | 4.88 |  |
|  |  |  |  |  |  |  |  | Both sexes |  | y |  | - |  | - |  | - |  | - |  | - |  | - |  | - |  | - |  | - |  | - |  | - |  | - |  | - |  | - |  | - |  | 2.36 |  | 1.2 |  | 4.66 |  |
| Metcalfe, C. |  | 2005 |  | Family economy conditions |  | N |  | male |  | y |  | - |  | - |  |  |  | - |  | - |  | - |  | - |  | - |  | - |  | - |  | - |  | - |  | - |  | - |  | - |  | 1.26 |  | 0.84 |  | 1.89 |  |
|  |  |  |  |  |  |  |  | male |  | n |  | - |  | - |  |  |  | - |  | - |  | - |  | - |  | - |  | - |  | - |  | - |  | - |  | - |  | - |  | - |  | 1.85 |  | 1.32 |  | 2.6 |  |
| Lawlor, D. A. |  | 2006 |  | Family economy conditions |  | N |  | male |  | n |  | - |  | - |  |  |  | - |  | - |  | - |  | - |  | - |  | - |  | - |  | - |  | - |  | - |  | - |  | - |  | 1.44 |  | 1.28 |  | 1.64 |  |
|  |  |  |  |  |  |  |  | female |  | n |  | - |  | - |  |  |  | - |  | - |  | - |  | - |  | - |  | - |  | - |  | - |  | - |  | - |  | - |  | - |  | 1.4 |  | 1.24 |  | 1.57 |  |
|  |  |  |  |  |  |  |  | Both sexes |  | y |  | - |  | - |  |  |  | - |  | - |  | - |  | - |  | - |  | - |  | - |  | - |  | - |  | - |  | - |  | - |  | 1.42 |  | 1.29 |  | 1.56 |  |
| Naess, O. |  | 2007 |  | Family economy conditions |  | N |  | Both sexes |  | y |  | - |  | - |  |  |  | - |  | - |  | - |  | - |  | - |  | - |  | - |  | - |  | - |  | - |  | - |  | - |  | 2.1 |  | 1.04 |  | 4.22 |  |
|  |  |  |  |  |  |  |  | Both sexes |  | n |  | - |  | - |  |  |  | - |  | - |  | - |  | - |  | - |  | - |  | - |  | - |  | - |  | - |  | - |  | - |  | 2.26 |  | 1.14 |  | 4.78 |  |
| Strand, B. H. |  | 2007 |  | Family economy conditions |  | N |  | male |  | n |  | - |  | - |  |  |  | - |  | - |  | - |  | - |  | - |  | - |  | - |  | - |  | - |  | - |  | - |  | - |  | 1.71 |  | 0.76 |  | 3.85 |  |
|  |  |  |  |  |  |  |  | male |  | y |  | - |  | - |  |  |  | - |  | - |  | - |  | - |  | - |  | - |  | - |  | - |  | - |  | - |  | - |  | - |  | 1.04 |  | 0.45 |  | 2.4 |  |
|  |  |  |  |  |  |  |  | female |  | n |  | - |  | - |  |  |  | - |  | - |  | - |  | - |  | - |  | - |  | - |  | - |  | - |  | - |  | - |  | - |  | 1.16 |  | 0.51 |  | 2.63 |  |
|  |  |  |  |  |  |  |  | female |  | y |  | - |  | - |  |  |  | - |  | - |  | - |  | - |  | - |  | - |  | - |  | - |  | - |  | - |  | - |  | - |  | 0.86 |  | 0.37 |  | 2 |  |
|  |  |  |  |  |  |  |  |  |  |  |  |  |  |  |  |  |  |  |  |  |  |  |  |  |  |  |  |  |  |  |  |  |  |  |  |  |  |  |  |  |  |  |  |  |  |  |  |
